# Coronavirus disease 2019 gastrointestinal and liver manifestations in adults: A review

**DOI:** 10.1002/jgh3.12671

**Published:** 2021-10-26

**Authors:** Apichet Sirinawasatien, Tanyaporn Chantarojanasiri, Sirina Ekpanyapong, Naris Tivatunsakul, Viravarn Luvira

**Affiliations:** ^1^ Division of Gastroenterology, Department of Medicine, Rajavithi Hospital, College of Medicine Rungsit University Bangkok Thailand; ^2^ Division of Gastroenterology, Department of Medicine Banpong Hospital Ratchaburi Thailand; ^3^ Department of Clinical Tropical Medicine, Faulty of Tropical Medicine Mahidol University Bangkok Thailand

**Keywords:** angiotensin‐converting enzyme 2 receptor, coronavirus disease 2019, diarrhea, gastrointestinal, liver, severe acute respiratory syndrome coronavirus 2

## Abstract

Coronavirus disease 2019 (COVID‐19) is an important health problem that has a serious adverse impact on the global economy and healthcare systems. The virus is not only involved in the respiratory system, but also causes other systemic effects as well as several gastrointestinal and liver issues. Evidence has shown direct viral invasion into the gastrointestinal tissue and supporting vascular network, causing various manifestations such as diarrhea, nausea, gastrointestinal bleeding, and abnormal liver function tests. The degree of gastrointestinal injury, especially in terms of liver involvement, is correlated with disease severity. There is no specific treatment for gastrointestinal involvement, and the symptoms can be managed with supportive therapy. Moreover, increased liver decompensation and mortality can be found in COVID‐19‐infected patients with coexisting liver disease. As the virus can be identified in gastrointestinal contents, endoscopic procedures during the pandemic should be carefully selected and proper protection strategies should be encouraged to prevent viral transmission.

## Graphical abstract

Patients with coronavirus disease 2019 typically present with fever and respiratory symptoms; however, gastrointestinal symptoms and liver issues have also been reported. Endoscopic procedures during the pandemic should be carefully selected and proper protection strategies should be encouraged to prevent viral transmission.

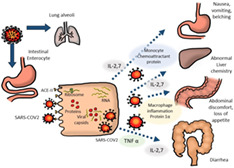

## Introduction

In December 2019, a group of patients in Wuhan, China, developed viral pneumonia caused by a newly identified β‐coronavirus.^1^, ^2^, ^3^ The virus was renamed, “severe acute respiratory syndrome coronavirus 2 (SARS‐CoV‐2),” and the disease that causes it is called coronavirus disease 2019 (COVID‐19).^2^, ^4^

SARS‐CoV‐2 is an enveloped, single‐stranded, positive‐sense RNA virus with a high transmission ability.^5^, ^6^ From the first outbreak on 30 January 2020, in China, COVID‐19 was registered as the sixth Public Health Emergency of International Concern (PHEIC) by the World Health Organization which declared COVID‐19 as a pandemic on 11 March 2020.^7^, ^8^ Later, SARS‐CoV‐2, spread all over the world, leading to more than 79.2 million cases and over 1.7 million deaths by the end of 2020.^9^

Gastrointestinal symptoms in COVID‐19 patients are common and have been reported to correlate with disease severity.^10^ Moreover, the virus particles can be identified in the gastrointestinal luminal content, which suggests a relationship between the virus and the gastrointestinal tract. This review aims to provide information regarding the gastrointestinal and liver manifestations of COVID‐19, as well as its management during pandemics. To achieve this, a search was made of English‐language human studies listed in the PubMed database, EMBASE, and other research published between February 2020 and March 2021. The keywords *gastrointestinal* and *liver* were used alone or in combination with *COVID‐19*. The references of the identified articles were also searched for potentially relevant studies, and systematic reviews, meta‐analyses, and case reports of special techniques were included. Duplicated data or data published as abstracts in academic meetings were excluded.

## Clinical course of SARS‐CoV‐2 infection

The mechanism by which a virus that originates in animals can spread to humans involves genetic alterations that enable it to infect and be transmitted from humans to humans. There is a similarity between SARS‐CoV‐2 and Bat‐CoV‐RaTG13 (a SARS‐like betacoronavirus in bats), supposing that a bat might be an initial host and *Manis pentadactyla* (Chinese pangolin) as the intermediate host, while humans act as accidental hosts. Some studies have shown that pigs or pangolins might have been intermediate hosts and snakes are probably among the virus reservoirs for human infection.^11^, ^12^

The incubation period for COVID‐19 is generally not greater than 14 days following exposure, with a median time of 5 days.^13^, ^14^ The infection is associated with five different clinical courses: Asymptomatic infection, mild to moderate cases, severe cases, critical cases, and death.^15^, ^16^ Although it is highly transmissible, more than 80% of infected patients have mild disease.^15^, ^17^ The remaining 20% have severe disease, and approximately 5% of patients exhibit critical illnesses such as respiratory arrest, septic shock, or multiple organ failure.^13^, ^18^ SARS‐CoV‐2 infection has an estimated 1–3% mortality rate due to the development of acute respiratory distress syndrome (ARDS), and uncontrolled immune stimulation, the so‐called “cytokine storm.” Risk factors associated with mortality include advanced age, obesity, diabetes, and hypertension.^19^ Other complications of COVID‐19 include cardiac and cardiovascular complications, arrhythmias, acute cardiac injury, and shock. Thromboembolic complications, including pulmonary embolism and acute stroke, as well as neurologic complications, including encephalopathy, have also been reported.

## Mechanism of gastrointestinal involvement

SARS‐CoV‐2 uses the receptor angiotensin‐converting enzyme 2 (ACE2) to enter cells in the human lower respiratory tract. This receptor is also abundant in gastrointestinal epithelial cells.^13^, ^20^, ^21^ As a result, apart from nasopharyngeal swabs, SARS‐CoV‐2 particles can also be found in fecal samples, esophagus, stomach, duodenum, and rectum.^22^, ^23^ Evidence of COVID‐19 infection in the gastrointestinal tract has also been discovered by isolating viral RNA from gastrointestinal epithelial cells and by intracellular staining of viral nucleocapsid proteins in the same cell.^24^

**Figure 1:**
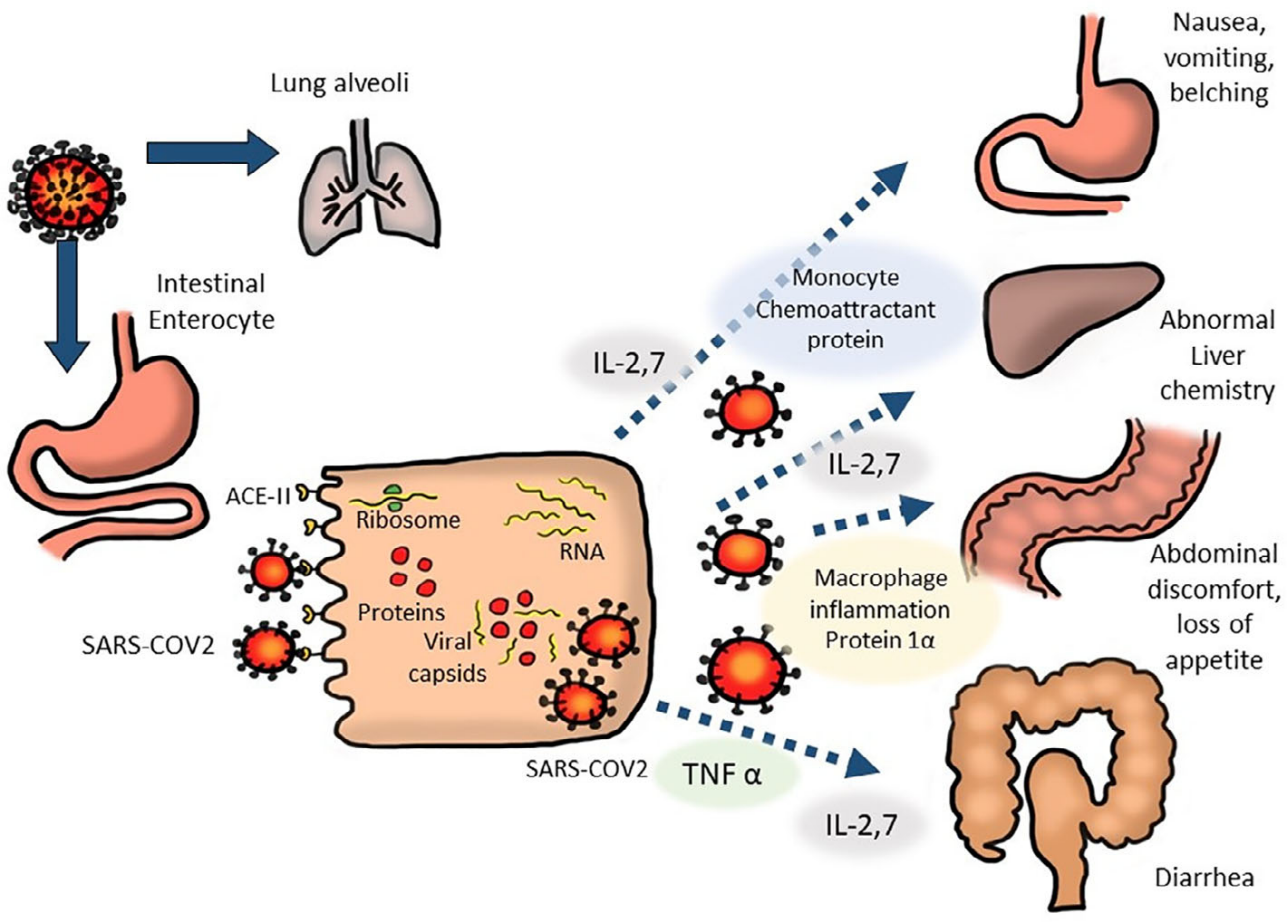
Schematic representation of the mechanism of coronavirus disease 2019 infection on the enterocytes and its effect with various part of gastrointestinal system. The virus adheres to the intestinal mucosa via angiotensin‐converting enzyme 2 receptors and produce various cytokines as well as chemoattractant proteins and inflammation proteins that cause injuries to various organ in gastrointestinal system (adapted from reference^[^ ^27^ ^]^).

The COVID‐19 pathogen enters the gastrointestinal epithelial cells through binding of its spike (S) proteins to the cellular surface ACE2 receptors (Fig. 1). Following cell entry, the virus hijacks host cell organelles to produce viral RNA and proteins. Finally, the newly assembled virions are secreted from the infected cell into the intestinal lumen by exocytosis.^25^ An intracellular interferon‐mediated immune response triggered by SARS‐CoV‐2 infection and the activation of immune responses from lymphocytes and inflammatory cells, which infiltrated the lamina propria, leads to the release of cytokines such as interleukin 2, 6, 7, 10, tumor necrosis factor (ΤΝF) α and calprotectin.^26^ These cytokines, in turn, mediate various effects on the gastrointestinal tract,^27^ as shown in Figure 1.

The SARS‐CoV‐2 viral RNA can be detected in feces in almost half (48.1%) of the COVID‐19 patients with gastrointestinal symptoms compared to approximately 9% of patients without gastrointestinal symptoms.^10^ The viral shedding in stool can persist up to 33–47 days after the first onset of illness, which is even longer than the clearance of the virus from the respiratory tract.^6^, ^28^, ^29^, ^30^ The prolonged duration of viral shedding through fecal material suggests the importance of preventive measures against fecal contamination.

There have been numerous reported cases of diagnosed COVID‐19 patients presenting with gastrointestinal manifestations, such as diarrhea, nausea, vomiting, and abdominal pain. The prevalence of GI symptoms varies greatly, ranging from 2 to 57%.^31^, ^32^, ^33^ In addition, many cases have been reported of abnormal liver chemistry during disease progression and higher rates of liver dysfunction have been found in patients with severe disease.^34^, ^35^, ^36^, ^37^, ^38^, ^39^, ^40^, ^41^

## Gastrointestinal manifestation

**Figure 2:**
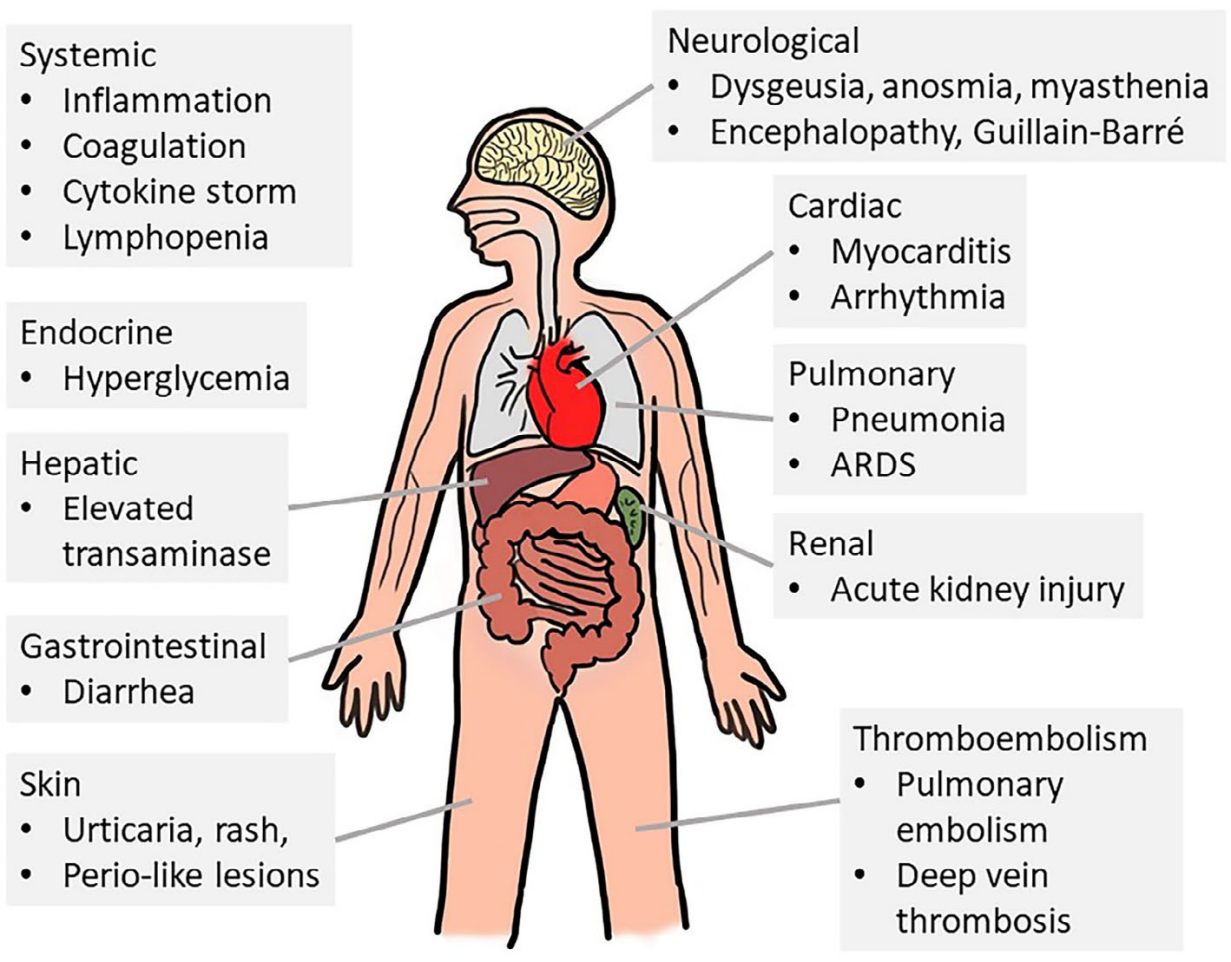
Systemic manifestations of coronavirus disease 2019 (adapted from reference^[^ ^19^ ^]^).

The symptoms of patients infected with SARS‐CoV‐2 are summarized in Figure 2.^19^ Several studies from different countries have reported a wide range of gastrointestinal symptoms (Table 1).^2^, ^42^ Also, these patients with gastrointestinal symptoms seem to require longer periods of hospitalization.^35^, ^36^, ^37^, ^43^ In contrast, the time from gastrointestinal symptoms to hospital presentation was 9 days, compared with 7.3 days for patients with respiratory symptoms.^13^

**Table 1:** Gastrointestinal findings in patients with coronavirus disease 2019

Ref.	Number of patients	Anorexia/Loss of appetite (*n*, %)	Diarrhea (*n*, %)	Nausea (*n*, %)	Vomiting (*n*, %)	Abdominal pain/discomfort (*n*, %)	GI bleeding (*n*, %)
Wang *et al*.^39^	138	55 (39.9%)	14 (10.1%)	14 (10.1%)	5 (3.6%)	3 (2.2%)	NA
Guan *et al*.^36^	1099	NA	42 (3.8%)	55 (5%) both for nausea and vomiting		NA	NA
Pan *et al*.^33^	103	81 (78.64%)	35 (33.98%)	NA	4 (3.88%)	2 (1.94%)	NA
Zhang *et al*.^83^	140	17/139 (12.2%)	18/139 (12.9%)	24/139 (17.3%)	7/139 (5%)	8/139 (5.8%)	NA
Lin *et al*.^84^	95 (58 with GI manifestations)	17 (17.9%)	23 (24.2%)	17 (17.9%)	4 (4.2%)	2 (2.1%)	2 (2.1%)
Cheung *et al*.^10^	59 (15 with GI manifestations)	NA	13 (22%)	NA	1 (1.7%)	7 (11.9%)	NA
Xia *et al*.^85^	20	NA	3 (15%)	NA	2 (10%)	NA	NA
Xiao *et al*.^24^	73	NA	26 (35.6%)	NA	NA	NA	10 (13.7%)

Gastrointestinal symptoms can be found ranged from 1.1 to 49.5% of COVID‐19 patients according to different studies. The most common symptoms are diarrhea (2–49.5%), anorexia (26.8%), nausea and or vomiting (3.9–10.2%), and abdominal pain (1.1–9.2%).^2^, ^10^, ^42^, ^44^, ^45^ Gastrointestinal symptoms usually worsen with disease progression and are correlated with a more insidious onset of disease.^2^, ^33^ There have also been reports of acute hemorrhagic colitis presenting with gastrointestinal bleeding.^2^, ^30^, ^33^, ^42^ Interestingly, in a meta‐analysis, the occurrence of gastrointestinal bleeding was found to be associated with increased mortality.^46^ Apart from the luminal involvement itself, endothelialitis and microthrombi with evidence of SARS‐CoV‐2 viral particle deposition have been reported in COVID‐19 patients presenting with respiratory failure, and nonocclusive mesenteric ischemia has been found in those who underwent colectomy.^47^, ^48^ These findings could be explained by the expression of ACE2 on intestinal enterocytes, which makes both the small and large intestines susceptible to SARS‐CoV‐2 infections.^24^, ^44^, ^49^, ^50^

Another organ that has been attacked by SARS‐CoV‐2 is the pancreas.^13^ A recent study by Wang *et al*. of 52 patients with COVID‐19 pneumonia revealed that 17% experienced pancreatic injury, which was defined as elevated amylase or lipase.^51^ Mechanisms by which pancreatic injury could occur include direct cytopathic effects of SARS‐CoV‐2, or indirect systemic inflammatory and immune‐mediated cellular responses, leading to organ damage or secondary enzyme abnormalities. Abundant amounts of ACE2 receptors are found in pancreatic islet cells, indicating that SARS‐CoV‐2 may also bind to ACE2 receptors in the pancreas and cause pancreatic injury.^52^

## Management of gastrointestinal involvements

Gastrointestinal symptoms such as nausea and vomiting can be treated with antiemetic medication; however, before initiating supportive care, further investigation is recommended to rule out infectious causes such as *Clostridium difficile* infection. The use of antibiotics remains controversial and is recommended only when a coinfection is noticed. Patients should be informed about hand hygiene and the importance of maintaining social distancing.^42^

Due to evidence of SARS‐CoV‐2 involving the gastrointestinal tract, many therapies and interventions for gastrointestinal diseases need to be adapted to reduce the spread of the virus through luminal content during the pandemic. There are several concerns and the recommended guidelines for endoscopic procedures, fecal transplantation, and management in patients with inflammatory bowel disease.

### Gastrointestinal endoscopy

As gastrointestinal endoscopy is considered to be an aerosol‐generating procedure,^53^ there have been many recommendations for gastrointestinal endoscopy during the COVID‐19 pandemic. The general recommendations are as follows^53^, ^54^:

1. Screening and assessment of the risk of COVID‐19 infection should be performed before endoscopy with consideration given to COVID‐19 screening before the procedure.
2. Elective procedures should be deferred.
3. The exposure of medical personnel should be minimized, and working schedules should be rearranged in accordance with local resources.
4. Proper use of personal protective equipment (PPE) needs to be practiced.
5. In cases of suspected or confirmed COVID‐19, endoscopy should be performed in a negative pressure room.
6. An enhanced disinfection policy for endoscopy rooms and reprocessing should be implemented.
7. Stepwise resumption of elective endoscopy should be managed according to local COVID‐19 controls and resources.

### Fecal transplantation

The fecal microbiota transplantation process also faces several challenges during the COVID‐19 pandemic. One of the most alarming problems is the possibility of COVID‐19 transmission from infected donors. Although most stool banks develop protocols for donor screening before fecal microbiota transplantation, the routine protocol may be unable to detect asymptomatic carriers. As a result, aggressive donor screening is recommended for all feces, regardless of risk factors.^44^, ^55^, ^56^

### Inflammatory bowel disease

Inflammatory bowel disease (IBD) is a chronic inflammatory disease that requires treatment with various immunomodulators and immunosuppressants. Moreover, many procedures may be required during treatment. In principle, the immunomodulators and immunosuppressants prescription should stop when patients test positive for SARS‐CoV‐2 unless there is an indication to use immunosuppressive agents (e.g. steroid, tofacitinib) for COVID‐19 treatment. If a patient with IBD is infected with SARS‐CoV‐2, treatment modification is recommended as follows^15^:

1. Patients who are taking 5‐aminosalicylic acid therapy should continue their treatment.
2. Patients taking budesonide therapy may continue their treatment.
3. Patients taking anti‐tumor necrosis factors should stop therapy.
4. Patients taking vedolizumab should stop therapy.
5. Patients on ustekinumab should stop therapy.
6. Patients taking prednisone ≥20 g/day should stop or taper the doses if possible.
7. Thiopurines (6‐mercaptopurine, azathioprine), methotrexate, and tofacitinib also tend to inhibit the body's immune response to viral infections; as a result, they should be stopped.

The above mentioned IBD medications can be restarted after patients recover from COVID‐19.^15^

## Liver manifestation of COVID‐19

Abnormal liver function is common in patients with COVID‐19. Aspartate transaminase (AST) or alanine transaminase (ALT) elevation has been reported in up to 13–58% of patients, while bilirubin elevation can be seen in 11–23% of patients, and less frequently, alkaline phosphatase (ALP) elevation in 5–10%, and gamma‐glutamyl transferase (GGT) elevation in 13–54% of patients.^35^, ^36^, ^57^, ^58^, ^59^, ^60^, ^61^, ^62^, ^63^, ^64^, ^65^ The pattern of liver injury is mostly hepatocellular rather than cholestatic and usually mild.^66^

Liver test abnormalities are more frequent in patients with more severe COVID‐19, and their severity correlates with the outcome of COVID‐19.^67^ A systematic review and meta‐analysis from China (35 studies, 6686 patients with COVID‐19) reported a significantly higher rate of abnormal liver function, including increased ALT (odds ratio [OR] = 1.89 [95% confidence interval—CI 1.30–2.76]; *P* = 0.0009) and increased AST (OR = 3.08 [95% CI 2.14–4.42]; *P* < 0.00001) in severe cases compared with nonsevere disease.^68^ Another study also demonstrated that 76.3% of patients with COVID‐19 (*n* = 417) had abnormal liver tests in hospital while 21.5% had “liver injury” defined as ALT and/or AST >3 × the upper limit of normal (ULN) or ALP, GGT, and/or total bilirubin >2 × ULN.^62^ AST is more frequently elevated than ALT and is associated with COVID‐19 severity and mortality, which might reflect immune‐mediated inflammation or other nonhepatic causes.^37^, ^69^, ^70^, ^71^ The presence of abnormal liver tests and liver injury was associated with progression to severe pneumonia, and the use of lopinavir/ritonavir was also found to increase the odds of liver injury by 4‐fold.^62^ In addition, low serum albumin levels on hospital admission were found to correlate with COVID‐19 severity.^69^, ^70^, ^72^

The possible pathogenesis of hepatic manifestation is believed to be multifactorial, including direct cytopathic effect of the virus, which may be related to the ACE2 receptor in the liver, hyper‐inflammatory cytokine and cytokine storm from immune responses, hypoxic–ischemic liver injury, drug‐induced liver injury, or coexisting with underlying liver diseases (e.g. chronic viral hepatitis, nonalcoholic fatty liver disease [NAFLD], cirrhosis).

## Chronic liver disease and cirrhosis with COVID‐19

Current studies reported that approximately 1.4–20% of patients infected with COVID‐19 also had concurrent chronic liver disease.^35^, ^36^, ^58^, ^62^, ^65^, ^70^, ^71^, ^73^ Recent data from two international registries (COVID‐Hep and SECURE‐Cirrhosis) on 745 COVID‐19 patients with pre‐existing liver diseases^74^ (including 359 chronic liver disease [CLD] without cirrhosis and 386 cirrhosis) demonstrated various etiologies of CLD, including 43% NAFLD, 24% alcoholic liver disease (ALD), 13% hepatitis B virus (HBV), 12% hepatitis C virus (HCV), and 6.4% with hepatocellular carcinoma (HCC). The presence of cirrhosis in CLD should be considered a risk factor for developing severe COVID‐19 and increased mortality rate (32% *vs* 8% when compared with no cirrhosis, *P* < 0.001).^74^ COVID‐19 infection has been reported to cause liver decompensation in one‐fifth of cirrhotic patients and worsen the status of liver decompensation or causes liver failure, especially in patients with diabetes and obesity.^75^, ^76^

In patients with cirrhosis, mortality increased according to the Child‐Pugh (CP) classification, with 19% in CP‐A, 35% in CP‐B, and 51% in CP‐C.^74^ The main cause of death was respiratory failure (71%). Factors associated with death were age (OR = 1.02; 95% CI 1.01–1.04), CP A (OR = 1.90; 95% CI 1.03–3.52), B (OR = 4.14; 95% CI 2.4–7.65), or C (OR = 9.32; 95% CI 4.80–18.08), cirrhosis and alcohol‐related liver disease (OR = 1.79; 95% CI 1.03–3.13).^74^ Moreover, emerging data have shown that NAFLD is associated with a higher risk of severe COVID‐19 and prolonged viral shedding time.^66^ Summarized studies that report the prevalence of liver test abnormalities in patients with COVID‐19, including those with CLD and cirrhosis, are described in Table 2.

**Table 2:** Prevalence of liver test abnormalities and mortality risk in patients with coronavirus disease 2019

Study	Country	Numbers	Pre‐existing liver diseases	AST elevation	ALT elevation	Bilirubin elevation	ALP elevation
Guan *et al*.^36^	China	1099	2.1%	22.2%	21.3%	10.5%	NA
Cai *et al*.^57^	China	298	9.4%	8.4%	13.1%	8.1%	0.3%
Fan *et al*.^58^	China	148	6.1%	21.6%	18.2%	6.1%	4.1%
Huang *et al*.^60^	China	36	NA	58.1%	13.3%	12.9%	NA
Cao *et al*.^61^	China	198	3%	17.4%	10.8%	2.6%	NA
Cai *et al*.^62^	China	417	5%	18.2%	12.9%	23.2%	4.8%
Zhang *et al*.^63^	China	115	NA	14.8%	9.6%	6.9%	5.2%
Tang *et al*.^64^	China	20 662 (meta‐analysis)	4.2%	23.6%	19.0%	9.5%	NA
Lei *et al*.^71^	China	5771	1.4%	Elevated AST was associated with the highest mortality risk			
Fu *et al*.^65^	China	482	19.9%	20.3%	19.9%	4.8%	NA
Ji *et al*.^66^	China	202	NAFLD 37.6% HBV 3.5%	16.8%	50%	8.4%	2.5%
Zhou *et al*.^77^	China	327	NAFLD 28.4%	The prevalence of severe COVID‐19 was observed in younger patients (age <60 years) with NAFLD more than 2‐fold higher than those without NAFLD			
Yadav *et al*.^78^	China	2115 (meta‐analysis)	4%	‐ High prevalence of liver injury (27%) ‐ Patients with liver injury had more severe disease and higher mortality ‐ Overall mortality in patients with COVID‐19 with liver injury 23.5%			
Sarin *et al*.^75^ (APCOLIS study)	13 Asian countries	228	185 CLD patients and 43 cirrhosis (NAFLD 55%, viral 30%)	Mortality in CLD patients with COVID‐19 *vs* cirrhosis with COVID‐19 (2.7% *vs* 16.4%, *P* = 0.002)			
Kulkarni *et al*.^67^	Multinational	20 874 (meta‐analysis)	CLD/Cirrhosis 61% NAFLD 19.5% HBV 17.8% HBV‐HCC 0.5% HCV 0.7%	‐ Pooled prevalence of CLD 3.6% (95% CI 2.5–5.1) ‐ Pooled incidence of elevated liver chemistries in COVID‐19 23.1% (95% CI 19.3–27.3) at initial presentation ‐ Pooled incidence of drug‐induced liver injury 25.4% (95% CI 14.2–41.4) ‐ COVID‐19 patients with elevated liver chemistries had increased risk of mortality (OR = 3.46 [95% CI 2.42–4.95], *P* < 0.001) and severe disease (OR = 2.87 [95% CI 2.29–3.6], *P* < 0.001) compared to patients without elevated liver chemistries			
Vespa *et al*.^79^	Italy	292	2%	18.5%	26.7%	10.6%	9.6%
Grasselli *et al*.^73^	Italy	1591	3%	Older patients (age ≥64 years) had higher mortality than younger patients (36% *vs* 15%; *P* < 0.001)			
Richardson *et al*.^80^	USA	5700	0.5%	58.4%	39.0%	Acute hepatic injury (AST or ALT >15 ULN) 2.1%	
Phipps *et al*.^70^	USA	2273	5%	56–74%	24–45%	‐ 45% mild, 21% moderate, 6.4% severe liver injury ‐ Peak ALT was significantly associated with death (OR = 1.14; *P* = 0.044)	
Hundt *et al*.^81^	USA	1827	NA (Obesity 42.5%)	66.9%	41.6%	4.3%	13.5%
Lavarone *et al*.^72^	Italy	50	Cirrhosis	67%	58%	‐ Overall 30‐day mortality rate of 34% ‐ COVID‐19 is associated with liver function deterioration and mortality in cirrhosis	
Bajaj *et al*.^82^	North America and Canada	‐ Patients with cirrhosis+COVID‐19 (*n* = 37) ‐ Patients with COVID‐19 (*n* = 108) ‐ Patients with cirrhosis (*n* = 127)	Cirrhosis	Patients with cirrhosis+COVID‐19 had higher mortality compared with patients with COVID‐19 (30% *vs* 13%, *P* = 0.03) but not between patients with cirrhosis+COVID‐19 and patients with cirrhosis (30% *vs* 20%, *P* = 0.16)			
Marjot *et al*.^74^ (SECURE‐cirrhosis and COVID‐Hep)	Multinational	745 ALD = 179 NAFLD = 322 HBV = 96 HCV = 92 HCC = 48	Chronic liver disease and cirrhosis	‐ Mortality in patients with cirrhosis 32% *vs* chronic liver disease 8% ‐ Mortality increased in Child‐Pugh class A (19%), B (35%), C (51%) ‐ ALD is an independent risk factor for death (OR = 1.79) ‐ NAFLD, viral hepatitis, and HCC have no independent association with death			

ALD, alcoholic liver disease; ALP, alkaline phosphatase; ALT, alanine transaminase; AST, aspartate transaminase; CI, confidence interval; CLD, chronic liver disease; HBV, hepatitis B virus; HCC, hepatocellular carcinoma; HCV, hepatitis C virus; NA, not available; NAFLD, nonalcoholic fatty liver disease; OR, odds ratio; ULN, upper limit of normal.

In summary, COVID‐19 causes a wide spectrum of gastrointestinal and liver involvement, ranging from direct invasion of the organism to the result of systemic immune processes. Gastrointestinal symptoms and liver function abnormalities are common during COVID‐19 infection and may reflect disease severity. The possibility of viral transmission through gastrointestinal content should be considered, and protection against infected luminal content and aerosol‐generated procedures during endoscopy should be emphasized. In addition, in patients with cirrhosis, COVID‐19 infection is associated with an increased risk of liver decompensation and increased mortality.

## Ethics approval

The study was approved by the ethics committee of Rajavithi Hospital.
